# Inhibition Role of Atherogenic Diet on Ethyl Carbamate Induced Lung Tumorigenesis in C57BL/6J Mice

**DOI:** 10.1038/s41598-017-05053-1

**Published:** 2017-07-05

**Authors:** Ting Chen, Lei Lu, Cai Xu, Xiaojing Lin, Yuet-kin Leung, Shuk-Mei Ho, Xiong Z. Ruan, Xuemei Lian

**Affiliations:** 1 Center for Lipid Research, Key Laboratory of Molecular Biology on Infectious Diseases designated by the Chinese Ministry of Education, Chongqing Medical University, Chongqing, China; 2 Department of Nutrition and Food Hygiene, School of Public Health and Management, Research Center for Medical and Social Development, Innovation Center for Social Risk Governance in Health, Chongqing Medical University, Chongqing, China; 3 Division of Environmental Genetics and Molecular Toxicology, Department of Environmental Health, University of Cincinnati College of Medicine, Cincinnati, OH USA; 4 John Moorhead Research Laboratory, Centre for Nephrology, University College London (UCL) Medical School, London, UK

## Abstract

With emerging evidence connecting cholesterol dysregulation with disturbed pulmonary homeostasis, we are wondering if diet induced hypercholesterolemia would influence the susceptibility to chemical induced lung tumorigenesis in mice. Six to eight week-old male C57BL/6J mice were fed with either a high-cholesterol atherogenic diet (HCD) or matching normal diet (ND), respectively. Following 3 weeks diet adapting, a multi-dose intraperitoneal injections of ethyl carbamate (urethane, 1 g/kg body weight) were established and lung tumorigenesis assessments were taken after 15 weeks latency period. Compared to the urethane treated ND-fed mice, the HCD-fed mice exhibited significantly decreased lung tumor multiplicity and attenuated pulmonary inflammation, which including reduced influx of leukocytes and down regulated tumor-promoting cyto-/chemokine profile in bronchoalveolar lavage fluid, decreased TLR2/4 expression and NF-κB activation in the lung. As a sensor regulating intracellular cholesterol homeostasis, nuclear receptor LXR-α was up-regulated significantly in the urethane treated HCD-fed mice lungs compared to the ND-fed mice lungs, accompanied with decreased pulmonary free cholesterol content and suppressed tumor cell proliferation. These results suggested that intrapulmonary cholesterol homeostasis, other than systematic cholesterol level, is important in lung tumorigenesis, and LXR activation might partly contribute to the inhibitory role of atherogenic diet on lung tumorigenesis.

## Introduction

Lung cancer is the most frequently diagnosed tumor and the most common cause of cancer mortality worldwide. The 2012 world cancer report (GLOBOCAN) shows that the world’s new cases of lung cancer has reached 1.8 million, 1.59 million of deaths, ranking first in the malignant tumor^1, 2^. Although smoking cessation is fundamental for lung cancer prevention, global statistics estimated that still 25% of lung cancer cases are not attributable to smoking^3^. Therefore, further studies are needed on the molecular pathways to modulate lung tumorigenesis/progression and on other strategies to prevent lung cancer.

Lipid metabolism disturbance has been connected to multiple cancer morbidity and mortality^4, 5^, but the association between cholesterol metabolism and lung cancer risk remains unclear. Majority of the epidemiologic studies documented lower total cholesterol levels in lung cancer patients, but the association was always interpreted as reverse causation, that is, undiagnosed cancer causing a reduction in cholesterol levels^6^. Still, there are studies found an association between low cholesterol and elevated lung cancer incidence among men even after excluding first 5–6 years of follow up and the prediagnostic cancer influence^7, 8^. Therefore, it has been difficult to entirely rule out the plausible relationship between low cholesterol levels and high lung cancer risk.

Recently, emerging evidences connecting cholesterol dysregulation with disturbed pulmonary homeostasis and chronic pulmonary inflammation were reported in several gene knockout mice. These genes are important in regulating cellular cholesterol metabolism. ABCA1 and ABCG1 are members of the ATP-binding cassette (ABC) family of transmembrane transporters involved in cellular cholesterol efflux^9^. Baldan’s study suggested that loss of ABCG1 resulted in severe pulmonary lipidosis and accompanied progressive chronic inflammation in the lungs of *abcg1* ^−/−^ mice^10^. Lysosomal acid lipase (LAL) plays a central role in the hydrolyzation of cholesteryl ester (CE) and triglycerides (TG) in the lysosomal of all cells^11^. Our previous work discovered that lal^−/−^ mice presented distinguishable lipidosis and progressive inflammation with abnormal recruitment of neutrophil and bronchoalveolar macrophages accumulation in the lung^11, 12^.

Since the link between cholesterol metabolism and lung cancer risk is undetermined and chronic inflammation and tumorigenesis is closely connected lately^13, 14^, it’s reasonable to speculate that the cholesterol metabolism disturbance could influence the susceptibility to lung tumorigenesis in mice. In this study, a commonly used high-cholesterol high-fat atherogenic diet induced hypercholesterolemia mouse model and a well-established multi-dose ethyl carbamate (urethane) induced C57BL/6J mice lung cancer model were used to test if diet induced hypercholesterolemia and disturbed lung integrity would influence the susceptibility to chemical induced lung tumorigenesis and tumor progression. Interestingly, the results showed that, compared to normal diet feeding, high-cholesterol high-fat diet feeding attenuated urethane induced lung inflammation and decreased multiplicity of lung tumorigenesis. Known as cellular “cholesterol sensor” and widely involved in modulating cholesterol metabolism, inflammation and cancer cell proliferation^15^, liver X receptor (LXR) activation partly contributed to the protection role of high cholesterol diet against urethane induced lung tumorigenesis in C57BL/6J mice.

## Results

### Influence of high cholesterol diet feeding on serum total cholesterol, whole body weight and fat mass in C57BL/6J mice

**Figure 1:**
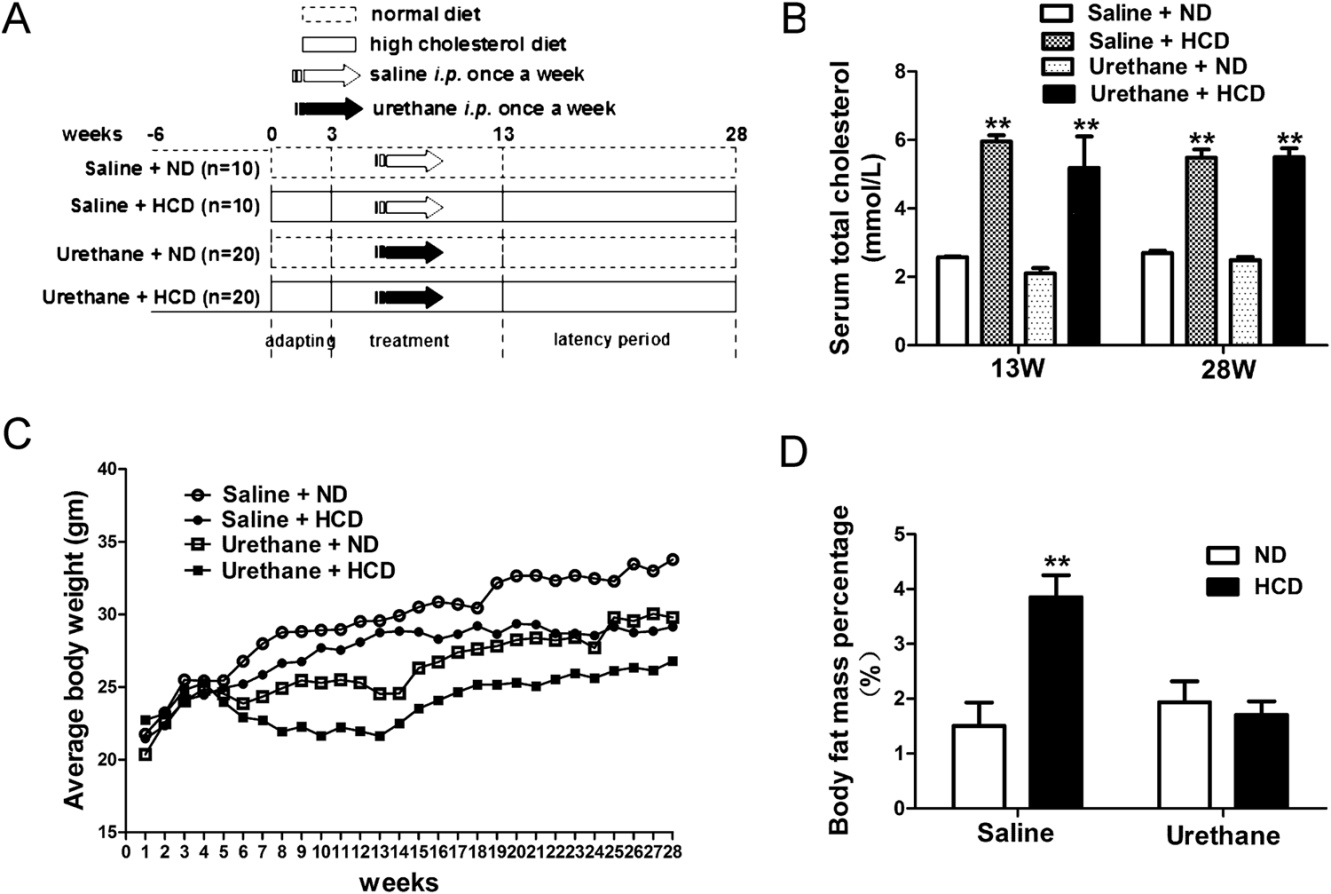
Differences of whole serum total cholesterol level, body weight and fat mass among study groups. (**A**) Schematic diagram of the experimental design for multiple urethane injections induced lung carcinogenesis model in C57BL/6J mice. (**B**) Serum total cholesterol levels analyzed using biochemical autoanalyzer at experimental week 13 and week 28 (n = 5 per group). Column, mean; bar, SEM. (**C**) Body weights of mice measured once a week (n = 10 per group). Dots indicate weight mean per group. (**D**) Quantification of whole body fat mass measured by NMR at experimental week 28 (n = 3 per group). Column, mean; bar, SEM. **P < 0.01 compared to ND fed mice with the same treatment. NMR: nuclear magnetic resonance; SEM: standard error of mean.

The high-cholesterol high-fat diet (HCD) adopted in this study was also called Paigen diet or atherogenic diet, which is widely used in the studies of atherosclerosis in C57BL/6J mice^16^. As expected, the HCD-fed mice developed significantly elevated serum total cholesterol compared to the matching normal diet (ND)-fed mice (Fig. 1B). Atherosclerotic plaques were also presented in aortas of 16 weeks HCD-fed mice (Supplemental Fig. 1). Consistent with previous studies^17^, obesity was not a phenotype of these HCD-fed mice, which showed modestly lower weight gain compared to ND-fed mice (Fig. 1C), despite the fact that daily food energy intakes were similar in both groups (data not shown). Interestingly, even though the HCD-fed mice had lower body weight, their body fat mass percentage was still higher than the ND-fed mice as shown by nuclear magnetic resonance data (Fig. 1D). Enlarged and fatty liver were presented in the HCD-fed mice (data not shown), indicating that lipid redistributions was presented during HCD feeding.

### Decreased lung tumor multiplicity in the urethane treated HCD-fed C57BL/6J mice compared to the ND-fed mice

**Figure 2:**
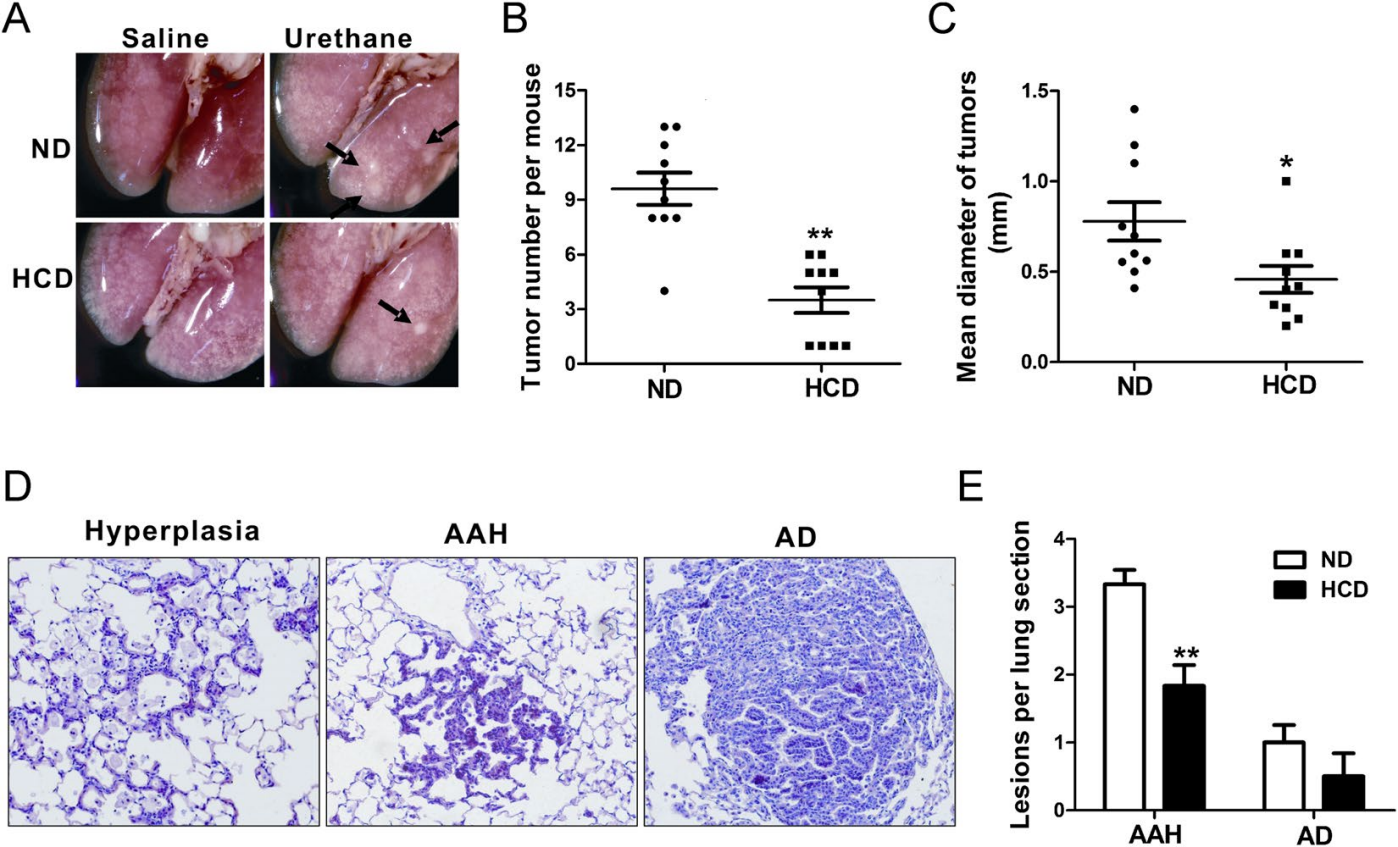
Decreased urethane induced lung tumor multiplicity and burden in HCD-fed C57BL/6J mice compared to ND-fed mice. Studies were carried out at experimental week 28, right after 15 weeks latency period. (**A**) Representative photographs of lungs under a dissecting microscope. Arrows point to visible lung tumors. (**B**,**C**) Tumor numbers and tumor diameters of urethane treated mice lungs (n = 10 mice per group). Dots indicate raw data points; lines, mean; bars, SEM. (**D**) Representative H&E staining of urethane treated mice lung sections, showing epithelial cell hyperplasia (Left), AAH (Middle) and AD (Right). (**E**) Numbers of different neoplastic lesion subtypes (AAH, AD) per mice (n = 6 per group). Columns, mean; bar, SEM. *P < 0.05, **P < 0.01 compared to ND-fed mice. AAH: atypical adenomatous hyperplasia; AD: adenoma.

It is known that mice with C57BL/6J background showed a strong resistance to lung tumorigenesis. Thus, to induce high incidence of lung tumors in C57BL/6J mice, a multiple urethane intraperitoneal (i.p.) injection protocol was established recently^18^. In our study, ten-weekly i.p. injection of urethane caused a 100% lung tumor incidence (number of mice with tumors/number of mice injected) in both HCD-fed and ND-fed C57BL/6J mice. While surprisingly, the multiplicity (number of tumors/mouse) and mean lung tumor diameters decreased significantly in the HCD-fed mice compared to the ND-fed mice (Fig. 2A~C). An extensive morphologic grading of lung neoplastic lesions revealed that atypical adenomatous hyperplasia (AAH), papillary and solid adenoma (AD) are the two main lesion types in these multidose urethane lung cancer models (Fig. 2D). No adenocarcinomas (AC) were observed. Fewer AAH and adenoma developed in the lungs of urethane treated HCD-fed mice compared to the ND-fed mice (Fig. 2E).

### Attenuated lung inflammation and NF-κB activation in the urethane treated HCD-fed C57BL/6J mice compared to the ND-fed mice

**Figure 3:**
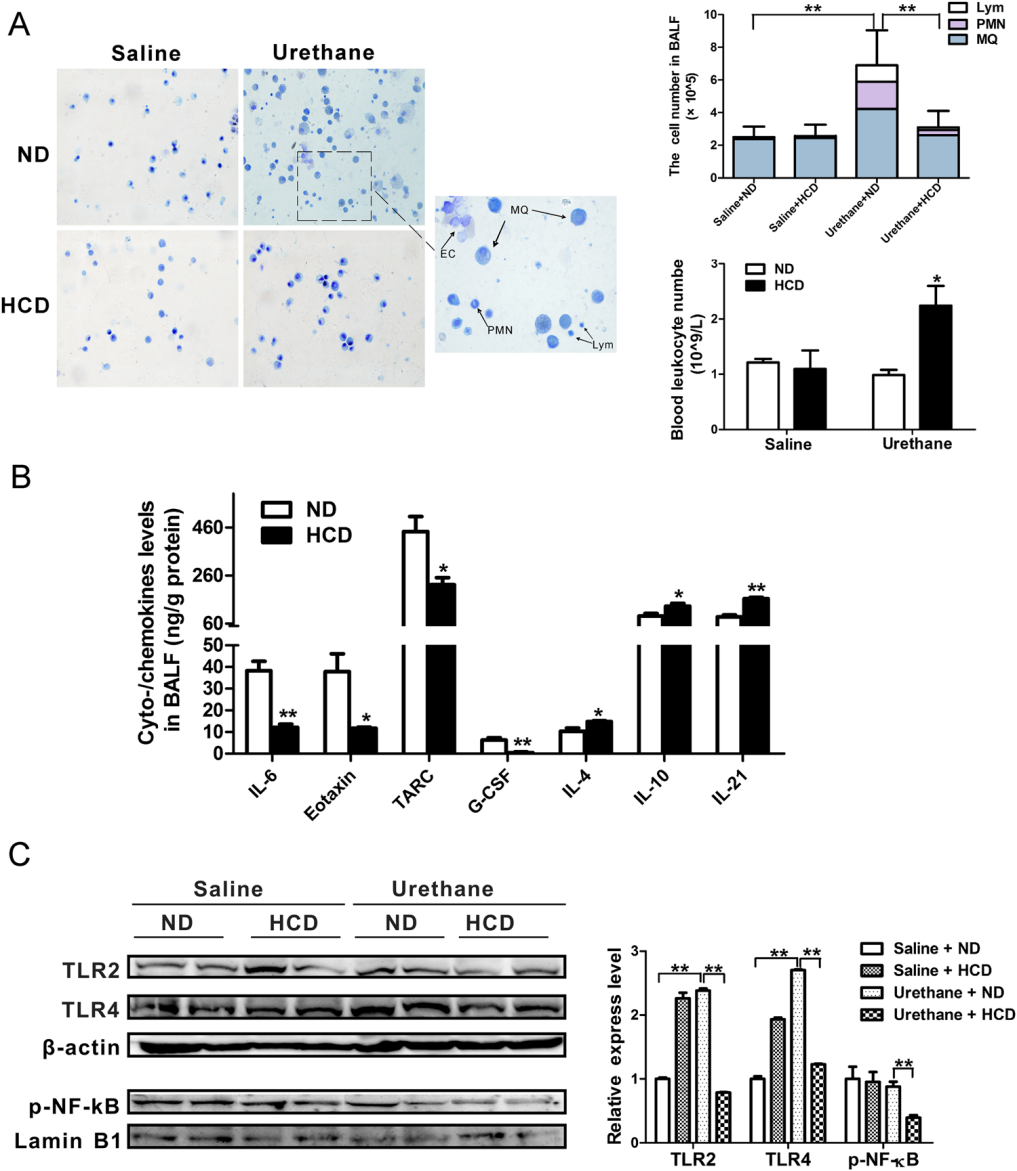
Attenuated lung inflammation and NF-κB activation in urethane treated HCD-fed C57BL/6J mice compared to ND-fed mice. Studies were carried out at experimental week 13, right after the 10 times consecutive saline or urethane injections. (**A**) Diff-Quik staining of BALF cells (left), BALF inflammatory cell counting (upper right), and blood leukocyte counting (lower right). n = 3–5 mice per group. (**B**) Multiplex antibody array quantification of selective inflammatory cyto-/chemokines in the BALF of urethane treated HCD-fed or ND-fed C57BL/6J mice (n = 3 mice per group). Cyto-/chemokines levels in BALF were corrected for BALF protein concentration and presented as ng/g protein. (**C**) Western blotting for the expression of TLR2, TLR4 and phosphorylated NF-κB p65 in the lungs. β-actin and Lamin B1 were used as the internal cytoplasma or nuclear protein control, respectively. Representative two mice results in each group and cropped gels/blots were displayed in the photos. Densitometry quantification was analyzed among 3 to 5 mice per group using Image J software. Columns, mean; bar, SEM. *P < 0.05; **P < 0.01. BALF: bronchoalveolar lavage fluid; MQ: macrophages; PMN: polymorphonucleated cells; EC: epithelial cells; Lym: lymphocytes.

Urethane-induced lung tumorigenesis is intimately associated with a lung inflammatory response^19^, so we have reasons to question whether the protective impact of HCD diet feeding would alter inflammatory parameters during the urethane-induced tumorigenesis in this study. In the ND-fed mice, urethane treatment significantly increased BAL immune cell infiltration compared with the normal saline treated controls (Fig. 3A). The urethane treated HCD-fed mice had significantly reduced influx of macrophage, neutrophil and lymphocyte into the BALF compared to the urethane-treated ND-fed mice as shown by Diff-Quik staining (Fig. 3A). Previous work demonstrated that dyslipidemia imparts opposing effects upon intra- and extra- pulmonary host defense^17^. Likewise, we found that circulation leukocyte numbers did increase mostly in the urethane-treated HCD-fed mice while the BALF inflammatory cell infiltration is less (Fig. 3A).

Expression levels of cyto-/chemokines in BALF showed significantly difference between urethane treated ND-fed mice and HCD-fed mice. Selected typical mediators involved in lung inflammation were presented in Fig. 3B. BALF levels of several important pro-inflammatory cyto-/chemokines like IL-6, CCL11 (eotaxin-1) and CCL17 (TARC) were significantly lower in BALF from the urethane treated HCD-fed mice compared to the ND-fed mice^20–22^. Conversely, IL-4, IL-10 and IL-21, mediators with known anti-inflammatory and immune surveillance functions^20, 23^, were up-regulated in BALF from the urethane treated HCD-fed mice.

Toll-like receptors (TLRs) are a family of transmembrane receptors that play key roles in both innate and adaptive immune responses and are involved in the development of such pathological conditions as infectious diseases, tissue damage, metabolic diseases and cancer^24^. Since lipid metabolites would be served as the endogenous ligands of TLRs, we are wondering if toll like receptor2/4 pathways were involved in the pulmonary inflammation of urethane treated mice. Correlated to the BALF leukocyte recruitment tendency, urethane treatment increased TLR2/4 expression in the ND-fed mice, while HCD feeding suppressed the urethane induced elevation of TLR2/4 expression (Fig. 3C). NF-κB, a central effector of inflammatory responses, has been identified as an important promoter of tumorigenesis in FVB and BALB/c mice models of lung adenocarcinoma^19^. In this study, NF-κB activation did not increase after urethane treatment in the lungs of the ND-fed C57BL/6J mice as reported^19^, whereas the expression of phosphorylated NF-κB p65 subunit in nuclear extracts of lung parenchyma significantly decreased in the urethane treated HCD-fed mice compared to the ND-fed mice (Fig. 3C).

### Elevated LXR activation in the urethane treated HCD-fed C57BL/6J mice compared to the ND-fed mice

**Figure 4:**
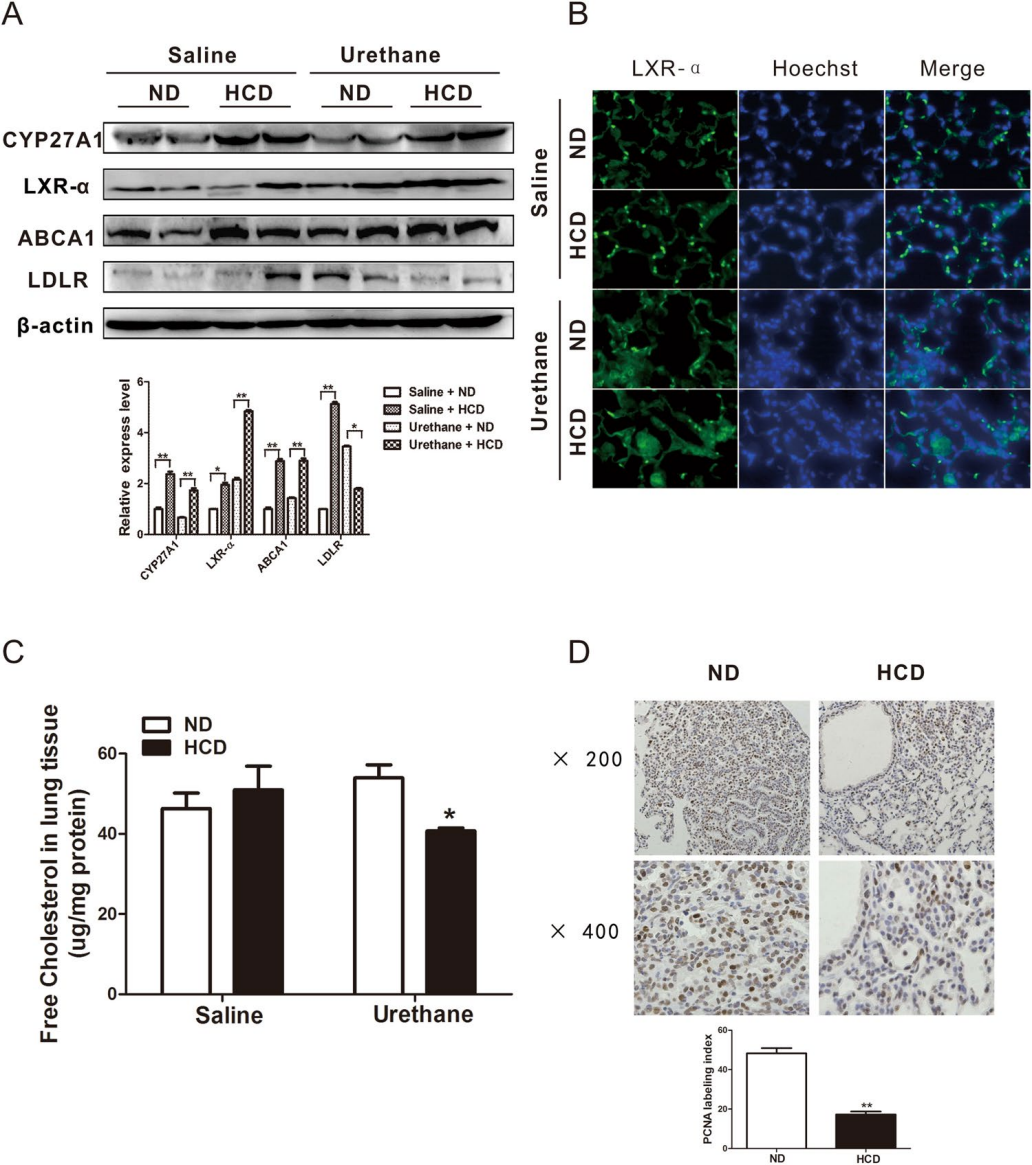
Elevated LXR activation in urethane treated HCD-fed C57BL/6J mice compared to ND-fed mice. (**A**) Western blotting for the expression of CYP27A1, LXR-α, ABCA1 and LDLR in the lung tissues collected at experimental week 13. Representative two mice results in each group and cropped gels/blots were displayed in the photos. Densitometry quantification was analyzed among 3 to 5 mice per group using Image J software. β-actin was used as the internal cytoplasma protein control. (**B**) Localization of LXR-α in the lung tissues through immunofluorescence techniques. green: LXR-α, blue: nucleus. original magnification: ×400. (**C**) Concentration of free cholesterol in lung tissues collected at experimental week 13 were measured with enzymatic assay (n = 3–5 per group). (**D**) Immunohistochemistry study of lung proliferation status using anti-PCNA antibody at experimental week 28. Dark brown color indicates the PCNA positive cells (original magnification: ×200; ×400). The PCNA labeling index was the percentage of PCNA-positive cells among total counted cells. n = 5 mice per group. Columns, mean; bar, SEM. *P < 0.05; **P < 0.01.

As liver X receptors (LXRs) are reported to regulate cholesterol metabolism and inflammation simultaneously^15^, and pulmonary cholesterol metabolites from sterol 27-hydroxylase (CYP27A1) pathway could act as endogenous ligand for LXR^25, 26^, we are wondering if LXR activation was involved in the protection role of HCD feeding in urethane induced lung tumorigenesis. Expectedly, the protein expression level of 27-hydroxylase and LXR-α increased significantly in steady-state HCD-fed mice compared to ND-fed mice, and urethane treatment further elevated the LXR-α expression in HCD-fed mice (Fig. 4A). The elevated LXR-α expression in HCD-fed urethane treated mice was also verified by the immunofluorescence localization of LXR-α expression in lung tissues (Fig. 4B). Two well-documented LXR target genes and readouts for LXR activation, ATP-Binding Cassette transporter A1 (ABCA1, a cellular cholesterol efflux mediator) protein expression levels markedly increased, and low density lipoprotein receptor (LDLR, a cellular cholesterol influx mediator) protein levels decreased in the urethane treated HCD-fed mice compared to the ND-fed mice (Fig. 4A). Consequently, free cholesterol contents in the lung markedly decreased in the urethane treated HCD-fed mice compared to the ND-fed mice (Fig. 4C).

LXR suppress the proliferation of a variety of human cancer cells *in vitro*, whose anti-proliferative effect has also been confirmed *in vivo* by evaluating the growth of human prostate cancer xenografts^27, 28^. In this study, attenuated lung tumor cell proliferations in the urethane treated HCD-fed mice compared to the ND-fed mice were visualized via PCNA immunohistochemical staining (Fig. 4D).

### Intranasal administration of T0901317 ameliorates urethane induced lung tumorigenesis

**Figure 5:**
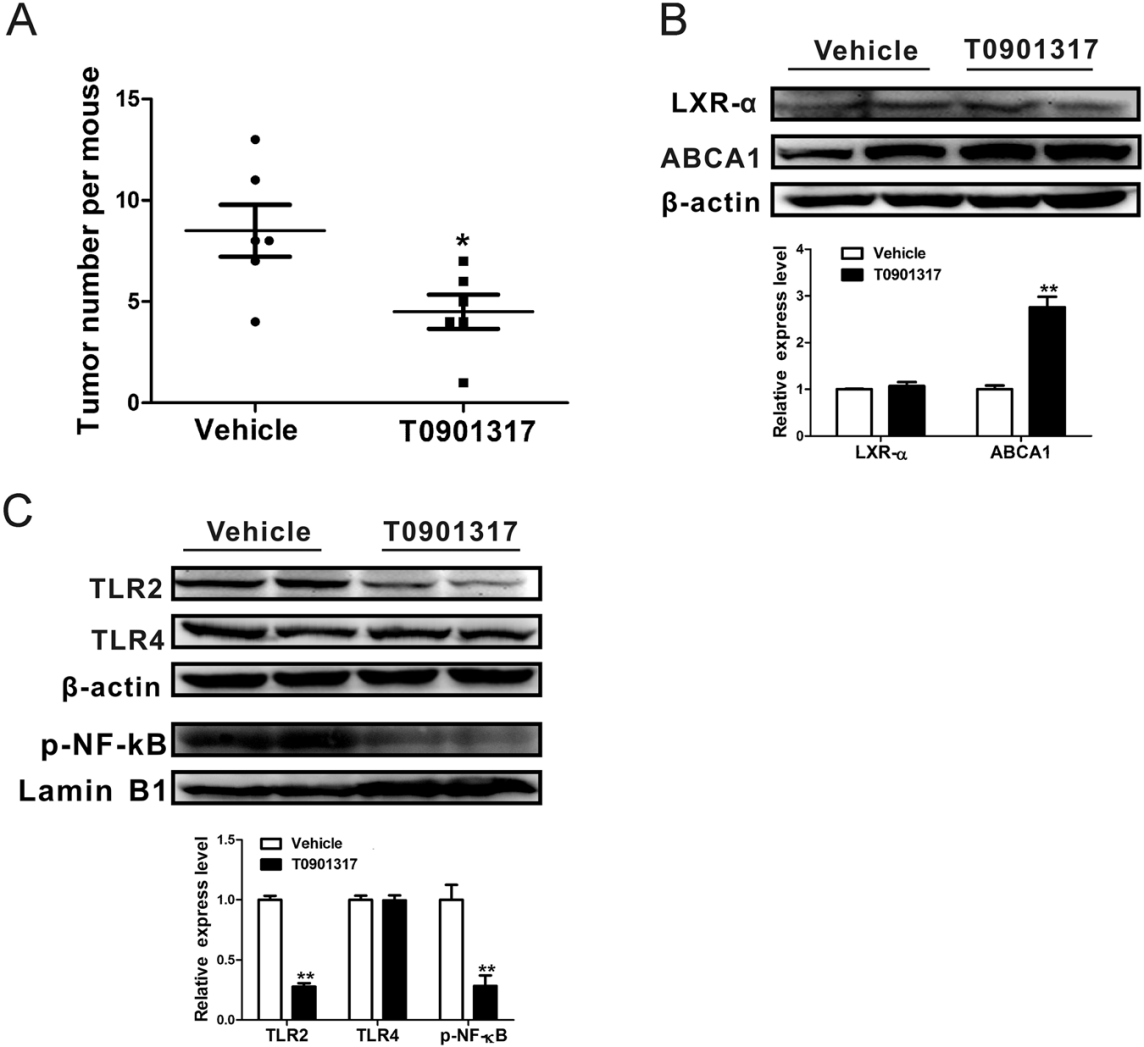
Intranasal administration of LXR agonist T0901317 attenuates urethane induced lung carcinogenesis in ND-fed C57BL/6J mice. (**A**) Tumor numbers in vehicle or T0901317 treated C57BL/6J mice lungs harvested at experimental week 28 (n = 6 per group). Dots indicate raw data points; lines, mean; bars, SEM. (**B**,**C**) Western blotting for the expression of LXR-α, ABCA1, TLR2, TLR4 and phosphorylated NF-κB p65 in vehicle or T0901317 treated C57BL/6J mice lung tissues collected at experimental week 13. β-actin and Lamin B1 were used as the internal cytoplasma or nuclear protein control, respectively. Representative two mice results in each group and cropped gels/blots were displayed in the photos. Densitometry quantification was analyzed among 3 mice per group using Image J software.

To further test the protection role of LXR activation in lung tumorigenesis, we investigated the effect of synthetic LXR agonist T0901317 administration in the preset multidose urethane induced lung tumorigenesis model. The ND-fed C57BL/6J mice were administrated 50 mg/kg T0901317 solution intranasal twice weekly during the 10-week period of urethane treatment, and DMSO treatment was used as the vehicle control. Consistent with our hypothesis, the multiplicity of lung tumor decreased significantly in the LXR agonist-treated mice compared to the vehicle control mice (Fig. 5A). Western blotting results showed that ABCA1 expression level remarkably up-regulated in the lungs of T0901317 treated mice (Fig. 5B) and verified the activation of LXR pathway. TLR2 and NF-κB expressions were attenuated as a consequence of LXR activation (Fig. 5C).

## Discussion

Due to the discrepancy of epidemiological studies and lack of relevant mechanism elucidated, the relationship between hypercholesterolemia and the risk of lung tumorigenesis is still not clear^6^. Given that oxysterols (the oxidation products of cholesterol) possess pro-oxidative and pro-inflammatory properties which can contribute to tumorigenesis^29^, it is surprising at first to find that high-cholesterol high-fat atherogenic diet feeding decreased the multiplicity of urethane induced lung tumorigenesis in this study. Further experiments confirmed that compared to ND-fed mice, urethane induced lung inflammation was also attenuated by HCD feeding, as the influx of inflammatory cells into the BALF was reduced, tumor-promoting cyto-/chemokine profile in BALF was down-regulated and TLR2/4 expression and NF-κB activation was also decreased. The protection role of the same atherogenic diet was also observed in urethane treated BABL/c mice and endotracheal orthotopic transplantation of A549 cell in nude mice model (data not shown).

Madenspacher JH *et al*. once used a similar high-cholesterol diet (HCD, 15.8 g% or 37.1 kcal% fat, with 1.25% cholesterol and 0.5% cholate added) to define whether diet induced hypercholesterolemia impacts responses to bacteria in the airspace^17^. They found that hypercholesterolemia imparts opposing effects upon intra- and extra-pulmonary host defense. Compared with ND-fed mice, HCD-fed mice had significantly reduced influx of leukocytes into the BALF following LPS inhalation, despite the relatively obvious circulating neutrophilia. The results exactly consistent to what we obtained in this research (Fig. 3A right). The authors proposed that airspace is a “privileged” site, which uniquely sensitive to hypercholesterolemia. In our study, we further elucidated that even though hypercholesterolemia were presented in HCD-fed mice (Fig. 1B), the intrapulmonary cholesterol contents decreased significantly in urethane treated HCD-fed mice compared to ND-fed mice (Fig. 4B). Therefore, intrapulmonary cholesterol homeostasis, other than systematic circulating cholesterol level, might play a surprisingly important and perhaps unique role in lung immune response and tumorigenesis. This could also partly explain why there are some contrasting effects of a high cholesterol diet upon growth of lung cancer and other cancer types like breast cancer^30^.

Cholesterol is a substantial (approximately 8%) but not the most essential component of pulmonary surfactant. To keep a physiologically low concentration of cholesterol in the lung, the cholesterol is continuously taken up by alveolar macrophages^31^. It has been demonstrated that sterol 27-hydroxylase (CYP27A1)-mediated reverse cholesterol transport is important in lung cholesterol homeostasis. CYP27A1 is a mitochondrial P450 enzyme able to further hydroxylate 27-OH cholesterol at the C-27 position, leading to the production of the carboxylic acid- cholestenoic acid. Cholestenoic acid has stronger polarity than un-metabolized cholesterol and is transported out of the cells more easily and independent of lipoprotein^32^. In the context of HCD induced hypercholesterolemia, pulmonary CYP27A1 expression increased in the HCD fed mice compared to the ND fed mice (Fig. 4A), suggesting a highly possibility that CYP27A1 mediated pulmonary cholesterol efflux might provide considerable amounts of metabolites for the activation of LXRs.

LXRs are best known as nuclear oxysterol receptors and physiological regulators of lipid and cholesterol metabolism^33^. LXR-β is ubiquitously expressed at a moderate level in most physiological systems, whereas LXR-α expression is mostly restricted to metabolically active tissues like liver, adipose tissue and lung etc. Both LXRs are regulators of multiple metabolic pathways, thus LXR activation may be highly beneficial as cellular cholesterol homeostasis depends on the integrated activities of the cholesterol uptake, efflux and synthesis pathways. LXRs induce expression of the E3 ubiquitin ligase IDOL (inducible degrader of LDLR) and the subsequent degradation of low density lipoprotein receptor (LDLR), which limits LDL cholesterol uptake in peripheral tissue cells^34^. LXRs also modulate ABCA1, transporter responsible for the export of intracellular cholesterol^33^. In this study, the elevated CYP27A1 expression and LXR activation, together with modulated ABCA1 and LDLR protein expression in the lung (Fig. 4A) might explain the phenotype of decreased intrapulmonary free cholesterol contents (Fig. 4B) and less cell proliferation (Fig. 4C) in the urethane treated HCD-fed C57BL/6J mice compared to the ND-fed mice.

Chronic inflammation is a characteristic phenotype of urethane induced lung tumorigenesis. LXRs were identified as anti-inflammatory transcription factors by considerable evidences. The first study linking LXRs to inflammatory responses was related to transcription nuclear factor NF-κB, which revealed that LXRs antagonized cytokine-mediated expression of pro-inflammatory genes as a consequence of transcriptional silencing of NF-κB^33^. Further extensive studies indicated that post-translational modification (PTM) could promote the transrepression function of LXR^35^. In this study, accompanied with LXR activation, down-regulation of TLR2/4 and NF-κB were simultaneously observed in the urethane treatment HCD-fed C57BL/6J mice compared to the ND-fed controls. Further LXR agonist T0901317 intranasal treatment study *in vivo* verified that LXR activation has a direct inhibitory effect on the expression of TLR2 and NF-κB (Fig. 5C).

Considering all the aforementioned evidences, we concluded that pulmonary LXR activation was accompanied by decreased pulmonary free cholesterol content, inhibited cancer cell proliferating, suppressed TLRs/NF-κB pathway related lung inflammation in urethane treated HCD-fed C57BL/6J mice in this study. Further LXR agonist T091317 ligand study verified that LXR activation could partly attenuate urethane induced lung tumorigenesis in C57BL/6J mice. Even though ablation of Liver X receptors α and β was reported to lead peripheral squamous cell lung cancer spontaneously in mice most recently^36^, we are aware that no previous reports have described a protection role of LXR activation against urethane induced lung cancer *in vivo*. Consistent with other study published recently^37^, the demonstrated ability of synthetic LXR ligand T0901317 to strengthen host resistance in this established chemical induced lung cancer model supports further exploration of LXRs as targets for lung cancer therapeutics.

In conclusion, atherogenic diet played a protection role on ethyl carbamate induced lung tumorigenesis in mice, which partly attributed to pulmonary LXR activation. Considering intrapulmonary cholesterol homeostasis, other than systematic cholesterol level, is most important in lung tumorigenesis, further studies exploring the specific pulmonary cholesterol metabolism pathways are critical to elucidate the possible biological association between lower cholesterol level and increased lung cancer risk in humans.

Given that the complex milieu during diet induced hypercholesterolemia, it is unlike that a single mediator is sufficient to interpret the protection role of HCD feeding in urethane induced lung tumorigenesis. Further studies to elucidate the effect of other cholesterol sensors like farnesoid X receptor (FXR), peroxisome proliferator-activated receptor-γ (PPAR-γ) are needed in the future.

## Methods

### Animals

Wild type C57BL/6J male mice were purchased from and kept in the laboratory animal facility of Chongqing Medical University. Mice were housed in a specific-pathogen free facility with a 12-hour light/12-hour dark cycle and were allowed unlimited access to sterilized chow and water. All animal care and experimental procedures were reviewed and approved by the Animal Care and Use Committee at Chongqing Medical University and all methods were performed in accordance with the relevant guidelines and regulations.

The mice body weight were monitored weekly and whole body fat mass were assessed right before the final end point (experimental week 28) using the quantitative nuclear magnetic resonance technique (Echo, MRI 3-in-1, Echo Medical Systems, Houston, TX). Blood leukocyte count was analyzed using the automated cytometry analyzer XE5000 (Sysmex Corporation, Shanghai, China). Serum total cholesterol levels were analyzed using a biochemical autoanalyzer (Roche, Miras, Switzerland).

### Experimental design

Six to eight week-old male C57BL/6J mice were randomly allocated to two groups, and placed on either a high-cholesterol high-fat diet (HCD) (20 g% or 40 kcal% fat, with 1.25% cholesterol, and 0.5% cholate added [D12109c; Research Diets Inc., New Brunswick, NJ]) or a matching normal diet (ND) (4 g% or 10 kcal% fat, with no cholesterol or cholate added, D12102c; Research Diets Inc.). The formula of the diets were presented in Supplemental Table 1. Following 3 weeks diet adapting, mice in each group were further randomly assigned to receive weekly intraperitoneal (i.p.) injections of either urethane (1 g/kg body weight in 100 μl saline) or normal saline control for 10 consecutive weeks, respectively. Lung tumorigenesis assessments were taken after 15 weeks latency period as described by York E. Miller and colleagues (Fig. 1A)^18^. Sets of mice (10–20 mice/group/time point) were sacrificed at experimental week 13 (after 10 consecutive i.p. injections of urethane or saline) or experimental week 28 (final end point) for further studies.

### Lung tumor enumeration

Mice were anesthetized and the lungs were resected after trans-tracheal inflation with 4% paraformaldehyde in phosphate-buffered saline (PBS). Surface lung tumor lesions were carefully counted by three blinded readers, and the lesion diameter was measured with the aid of digital calipers under a dissecting microscope, as described by Singh and colleagues^38^.

### Histology and immunohistochemistry assay

The assay followed a procedure published previously^11^. Briefly, Excised mouse lungs were inflation-fixed in 4% neutral buffered paraformaldehyde overnight at 4 °C before proceeding to paraffin embedding. Five-μm-thick sections were mounted on glass slides and stained with hematoxylin and eosin (H&E). The proportion of each type of lung lesions, including atypical adenomatous hyperplasia (AAH), adenoma, and adenocarcinoma were evaluated on sections from each lung and results were averaged per mouse^39^. For immunohistochemical (IHC) study, sections were incubated with goat anti-mouse PCNA antibody (1:100; Epitomics, San Diego, CA) as the primary antibodies. Quantification of PCNA positive staining was determined by counting 10 fields for each lung section at ×400 amplify using the method described by Kobayashi N *et al*.^40^, and the average positive count was expressed as a percentage.

### Bronchoalveolar lavage and multiplex antibody array

Bronchoalveolar lavage was performed using three aliquots of 1 mL sterile normal saline as previously described^11^. Bronchoalveolar lavage fluids (BALF) were centrifuged at 400 g for 10 minutes to separate cells from supernatants. Total cell numbers was determined using a grid hemocytometer. Cell suspension was subjected to cytospin and stained with Diff-Quik stain kit (Yuanye, Shanghai, China) for the assessment of BALF inflammatory cell types. The BALF supernatants were used in multiplex antibody array for cyto-/chemokine measurements. 40 cytokines and chemokines were determined via glass slide-based quantitative antibody arrays (Quantibody^@^ Mouse Inflammation Array 1, Raybiotech Inc., Norcross, GA). Cyto-/chemokine levels were corrected for BALF protein concentration and presented as nanograms per gram of protein.

### Isolation of proteins and western blot analysis

Before lung excision, the right ventricle was infused with at least 20 ml of sterile 0.9% saline to remove any residual blood in the pulmonary vasculature. Lung tissues were lysed using either nucleoprotein or cytoplasm protein extraction kit (KeyGEN BioTECH, Nanjing, China), respectively. Protein extracts were analyzed on sodium dodecyl sulfate–polyacrylamide gel electrophoresis (Bio-Rad, Hercules, CA). Primary antibodies used in the study were: Anti- phospho-NFκB p65 (Cell Signaling, Boston, MA), anti-Lamin B1 (Bioworld, Louis Park, MN), anti-β-actin (Zhongshan, Beijing, China), anti-ABCA1 (Santa Cruz Biotechnology, CA), anti-TLR2/4, anti-CYP27A1, anti-LXR-α and anti-LDLR (Abcam, Hongkong, China).

### Immunofluorescence

Sliced lung tissue samples were heated at 60 °C for about 30 minutes, then deparaffinized with xylene and series of ethanol. Next, antigen retrieval of tissue samples was performed by incubating tissues with pre-warmed citrate buffer (pH = 6.0), then cooled and washed with ddH2O and PBS. To inactivation of endogenous peroxidase, antigen retrieved tissues were incubated with 3% hydrogen peroxide for 15 min, and then washed with PBS three times. Subsequently, samples were then blocked with normal goat serum for 30 min. Tissue slides were incubated with LXR-α antibodies (1:200 dilution, Abcam, Hongkong, China) overnight at 4 °C. After washing three times with PBS, tissue slides were incubated with FITC-conjugated secondary antibody (1:200 dilution) and Hoechst 33258 for 30 min at 37 °C. Finally, tissue slides were washed with PBS three times, mounted with anti-fluorescence quenching liquid. Images were obtained by using a CKX41 microscope (Olympus). Fluorescence intensity of protein was analyzed by using Image J software (NIH, Bethesda, MD, USA).

### Quantitative measurement of lung tissue cholesterol

Free cholesterol levels in lung tissues were measured by an enzymatic assay described by Robinet P *et al*.^41^. In brief, 1 mL hexane: isopropanol (3:2, v- v) was added to the homogenized lung tissue for lipid extraction. After 1 min sonication, the lipid phase was collected and dried in vacuum. Dried lipid phase was then dissolved in isopropanol: NP40 (9:1, v- v). Concentrations of free cholesterol were analyzed using a standard curve and normalized by total protein from the lung tissues.

### LXR ligand treatment

The same multidose urethane induced lung cancer model was applied in normal diet fed wild type C57BL/6J mice. Simultaneously, the mice were intranasal administrated with either vehicle (DMSO) or T0901317 (Purity ≥ 98%, Cayman Chemicals Co., Ann Arbor, MI; 50 mg/kg body weight in 20 μl DMSO) twice a week for 10 consecutive weeks, respectively. Lung tumorigenesis assessments were taken after 15 weeks latency period.

### Statistical analysis

Results are expressed as mean ± SEM. To compare means between groups, Student’s t test or two-way analysis of variance (ANOVA) with LSD post hoc tests were used. All P values were 2-tailed. *P* values < 0.05 were considered significant. Statistical analyses were performed by using the Statistical Package for the Social Sciences Software Version 11.0 (SPSS, Chicago, IL).

## Electronic supplementary material

Supplemental data
