# Supplementary material for: Inhibition Role of Atherogenic Diet on Ethyl Carbamate Induced Lung Tumorigenesis in C57BL/6J Mice
Source: Sci Rep. 2017 Jul 5;7:4723. doi: 10.1038/s41598-017-05053-1 (PMC5498653; doi:10.1038/s41598-017-05053-1)
Supplement: Supplementary file 1 — Supplemental data [file 41598_2017_5053_MOESM1_ESM.pdf]

Inhibition Role of Atherogenic Diet on Ethyl Carbamate Induced Lung Tumorigenesis in C57BL/6J Mice

Ting Chen[1,2,#], Lei Lu[1,2,#], Cai Xu[1,2], Xiaojing Lin[1,2], Yuet-kin Leung[3], Shuk-Mei Ho[3], Xiong Z Ruan[1,4] and Xuemei Lian[1,2,*]

Supplemental Table 1 Diet Formulations

Diet component	RD		HCD	
	g%	kcal%	g%	kcal%
Protein(casein, L-cysteine)	19.2	20	22.5	20
Carbohydrate (corn starch, maltodextrin 10, sucrose, cellulose)	67.3	70	45	40
Fat (cocoa butter, soybean oil)	4	10	20	40
Cholesterol	0	0	1.25	0
Sodium cholate	0	0	0.5	0
Vitamin, mineral, others	~10	0	~10	0

RD: regular diet; HCD: high cholesterol/high fat diet

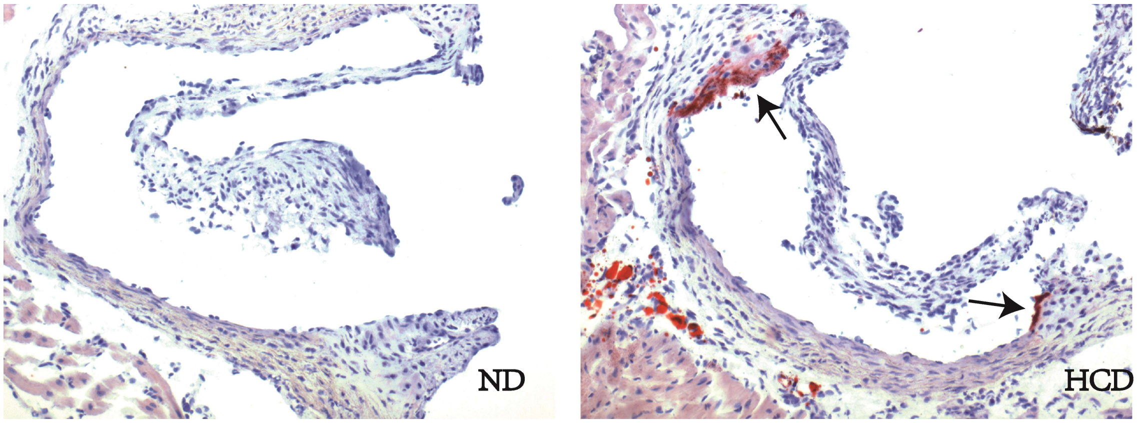

Supplemental Figure 1 Oil red O staining of aortas after 16 weeks feeding of either normal diet (ND) or high fat/high cholesterol diet (HCD) in C57BL/6J mice. Aortas frozen sections were prepared and atherosclerotic plaques (arrows) were visualized by Oil Red O staining. (original magnification: ×100)
